# A comprehensive flow-cytometric analysis of graft infiltrating lymphocytes, draining lymph nodes and serum during the rejection phase in a fully allogeneic rat cornea transplant model

**Published:** 2011-02-08

**Authors:** Martin Maenz, Mourice Morcos, Thomas Ritter

**Affiliations:** College of Medicine, Nursing and Health Sciences, Regenerative Medicine Institute, National Centre for Biomedical Engineering Science, National University of Ireland, Galway, Galway, Ireland

## Abstract

**Purpose:**

To establish a cornea transplant model in a pigmented rat strain and to define the immunologic reaction toward corneal allografts, by studying the cellular and humoral immune response after keratoplasty.

**Methods:**

Full thickness penetrating keratoplasty was performed on Brown Norway (RT1n) recipients using fully major histocompatibility complex (MHC)-mismatched Piebald-Viral-Glaxo (PVG; RT1c) donors. Using multicolor flow cytometry (FACS) we quantified and compared the cellular composition of draining versus non-draining lymph nodes (LN). Furthermore, we developed an isolation method to release viable graft infiltrating lymphocytes (GIL) and subjected them to phenotypic analysis and screened serum from transplanted animals for allo-antibodies.

**Results:**

Assessing ipsi-lateral submandibular LN we find ample evidence for post surgical inflammation such as elevated absolute numbers of cluster of differentiation (CD)4^+^, CD8^+^, B-cells, and differential expression of CD134. However, we could not unequivocally identify an allo-antigen-specific immune response. FACS analysis of lymphocytes isolated from collagenase digested rejected corneas revealed the following six distinct subpopulations: MHC-2^+^ cells, CD4^+^ T-cells, CD8^+^ T-cells, CD161^dull^ large granular lymphocytes, CD3^+^ CD8^+^ CD161^dull^ natural killer (NK)-T-cells and CD161^high^ CD3^-^ NK cells. At post-operation day (POD)-07 only CD161^dull^ MHC-2^neg^ large granular lymphocytes (LGLs) were detected in syngeneic and allo-grafts. In concordance with an increase in B-cell numbers we often detected copious amounts of allo-antibodies in serum of rejecting animals, in particular immunoglobulin (Ig) M (IgM), immunoglobulin (Ig) G1 (IgG1), and IgG2a.

**Conclusions:**

Our results demonstrate that despite its immune privileged status and low-responder characteristics of the strain combination, allogeneic corneal grafts mount a full fledged T helper1 (Th1) and Th2 response. The presence of NK-T-cells and NK-cells in rejecting corneas shows the synergy between innate and adaptive immunity during allograft destruction.

## Introduction

Animal models of penetrating keratoplasty have been valuable research tools for our understanding of allo-rejection processes in the context of an immune privileged site [1]. The cellular key players and the corner stones of the rejection pathways have been elucidated [2]. The cervical lymph nodes (LN) have been unequivocally identified as the location where the allo-recognition of corneal grafts is concentrated [3-6]. So far indirect in-vitro methods have been used to identify specific T-cell responses mounted against allogeneic corneal transplants [7]. We hypothesized that by adopting a multi-parameter flow cytometry (FACS) approach to both identify and quantify lymphocyte populations in the draining lymph nodes and to screen for T-cell activation markers, it would be possible to directly assess allo-reactive T-lymphocytes and define the characteristics of our transplant model. We specifically chose a rarely used low responder model to study the rejection process [8] and sought to determine whether previous results from high responder strain combinations such as Lewis-Brown Norway (LEW-BN) or Lewis-Dark Agouti (LEW-DA) can be reproduced.

Of particular interest to us was the determination of graft infiltrating lymphocytes. In the past immuno-histochemistry (IHC) was the method of choice to identify the different immune cells [9-11]. However, IHC is difficult to establish and in general laborious, effectively limiting the number of samples processed and the application of multi-parameter analysis. Additionally, results are often difficult to interpret and are prone to subjective bias. Instead, we developed a digestion procedure, which releases viable cells from corneal tissue. To demonstrate the effectiveness of this novel approach we used multicolor FACS to describe the cells involved in the graft destruction process.

## Methods

### Animals

All procedures performed were conducted under animal license number B100/3852 and were approved by the Animals Ethics Committee of the National University of Ireland, Galway. In addition, animal care and management followed the Standard Operating Procedures of the Animal Facility at the National Centre for Biomedical Engineering Science. Brown Norway (BN, RT1n) and Piebald-Viral-Glaxo (PVG, RT1c) rats were purchased from Harlan Laboratories UK and housed under specific pathogen free conditions with food and water ad lib.

### Keratoplasty model

A low-risk fully allogeneic major histocompatibility complex-1 (MHC-1/MHC2 and non-classical MHC) with BN as recipient and PVG as donor was established. All animals were male and of 8–14 weeks age.

### Anesthesia

Isoflurane was administered systemically at 2%–2.5% in medical oxygen (BOC, Galway, Ireland) with a flow rate of 2 l/min. Local anesthesia was performed with Tetracaine 1% (Chauvin Pharmaceuticals Ltd., Kingston-upon-Thames , UK). Iris dilation was performed with Atropine 1%, Tropicamide 1% and Phenylephrine 2.5% (all Chauvin Pharmaceuticals Ltd.). A 3 mm full thickness graft was placed on a 2.5 mm graft bed, fixed with 8–10 interrupted 10–0 Ethilon® sutures (Ethicon, Livingston, Scotland) and covered with chloramphenicol antibiotic ointment. Alcon BSS® (Alcon, Hemel Hempstead, UK) was used for irrigation of cornea tissue. Eyelids stayed open post-op and the sutures were not removed. Graft appearance was assessed every other day and the opacity graded according to the following scale modified for pigmented iris: 0-no opacity; 1-minimal-all iris details (crypts) visible; 2-some iris details visible; 3 strong-only pupil margin visible; 4 complete-anterior chamber not visible. An opacity ≥3 was considered rejected.

### Flow-cytometry

Information on primary antibodies and appropriate isotype controls are presented in Table 1. Staining was performed according to standard protocols including Fcγ-block, live/dead stain (violet Live/Dead; Invitrogen, Dun Laoghaire, Ireland) and endogenous biotin blocking (Molecular Probes (division of Invitrogen) Dun Laoghaire, Ireland). Flowcytometric data was acquired and analyzed according to principles laid out in detail by Herzenberg et al. [12].

**Table 1:** Antibodies used for Flow-cytometry.

**Name**	**Isotype**	**Clone**	**Host**	**Label**	**Supplier code**	**Supplier**
CD3	IgG3aκ	G4.18	mouse	PE	554833	BD Biosciences
CD3	IgG3aκ	G4.18	mouse	FITC	11-0030-82	eBioscience
CD4	IgG2aκ	Ox-35	mouse	APC	17-0040	eBioscience
CD8	IgG1	Ox-8	mouse	Biotin	MCA48B	Serotec
CD8	IgG1	Ox-8	mouse	AF647	MCA48AF647	Serotec
CD25	IgG1	Ox-39	mouse	FITC	554865	BD Biosciences
CD32 Fc-γ Block	IgG1	D34-485	mouse	NALE	550271	BD Biosciences
CD45RA	IgG1	Ox-33	mouse	FITC	MCA430F	Serotec
MHC-2	IgG1	Ox-6	mouse	PE	MCA46PEB	Serotec
CD134	IgG1	Ox-86	mouse	PE	MCA1420PE	Serotec
CD161	IgG1	10/78	mouse	PE	555009	BD Biosciences
CD161	IgG1	10/78	mouse	Biotin	MCA1427B	Serotec
IgM	IgG1	G53-238	mouse	PE	553888	BD Biosciences
**Isotype control antibodies**						
Isotype control	IgG1	F8-11-13	mouse	Biotin	MCA1209B	Serotec
Isotype control	IgG1	F8-11-13	mouse	PE	MCA1209PE	Serotec
Isotype control	IgG1	F8-11-13	mouse	AF647	MCA1209A647	Serotec
Isotype control	IgG1	MOPC-21	mouse	FITC	555909	BD Biosciences
Isotype control	IgG2aκ	-	mouse	APC	17-4724	eBioscience
Isotype control	IgG3aκ	A112-3	mouse	PE	559929	BD Biosciences
Isotype control	IgG3aκ	B10	mouse	FITC	11-4742-73	eBioscience
**Secondary reagents**						
Sav-PerCP-Cy5.5					551419	BD Biosciences
Sav-PE-Cy7					557598	BD Biosciences

### Isolation of lymphocytes from draining LN and corneal tissue

Lymph nodes and corneas were digested with 5% w/v Collagenase D (Roche Diagnostics Ltd, Burgess Hill, UK) in RPMI containing 25 mM HEPES (Lonza Wokingham, Ltd., Berkshire, UK) plus 1% fetal calf serum (Sigma-Aldrich Ireland Ltd.; Dublin, Ireland) at 37 °C. Cornea specimens were agitated on an Eppendorf Thermomixer^®^ R (Eppendorf UK Limited, Cambridge, UK) for 90 min at 900 per minute mixing frequency during the digestion reaction. LNs were incubated for 30 min under the same conditions. The digestion reaction was stopped by adding an excess volume of cold PBS+2mM EDTA (Lonza Wokingham, Ltd.). Remaining undigested tissue was carefully poured into a 100 µM cell strainer and disintegrated with the plunger of a syringe. Cell suspensions were collected in 6 cm tissue culture dishes then transferred into 15 ml tubes spun at 400× g for 4 min and washed again with PBS+2 mM EDTA. Individual cornea preparations were used for subsequent multi-color FACS stainings.

### Serum analysis

Blood was collected by cardiac puncture and left to coagulate for serum harvesting. Diluted serum (1:5) was incubated with Piebald-Viral-Glaxo (PVG) splenocytes for 30 min and then stained with either anti-rat IgM [G53–238] or IgG1 [MRG1–58] or IgG2a [MRG2a-83] or IgA [MARA-1] (all from Antibodies-online GmbH, Aachen, Germany). Positive control sera were generated by immunising naïve BN rats with 1×10^6^ γ-irradiated (12 Gy) thymic dendritic cells. Thymic dendritic cells (DCs) were obtained by density gradient centrifugation (14.5% w/v Nycodenz® [Axis-Shield UK, Cambridgeshire, UK]) according to the method published by Milling et al. [13]. Positive sera were harvested 14 days post immunization.

### Flow-cytometry equipment and software

FACSAria-1, FACSAria-2 and FACSCanto (BD Biosciences, Oxford, UK); Acquisition software was FACSDiva V5 and V6 (BD Biosciences). Analysis software was Flowjo V7.6 for PC, (Treestar, Inc., Ashland, OR).

### Statistics

Non-parametric Mann–Whitney-U test was used throughout the statistical analysis. A p-value ≤0.05 was deemed statistically significant and indicated where appropriate.

## Results

### General characteristics of the BN-PVG model

**Figure 1:**
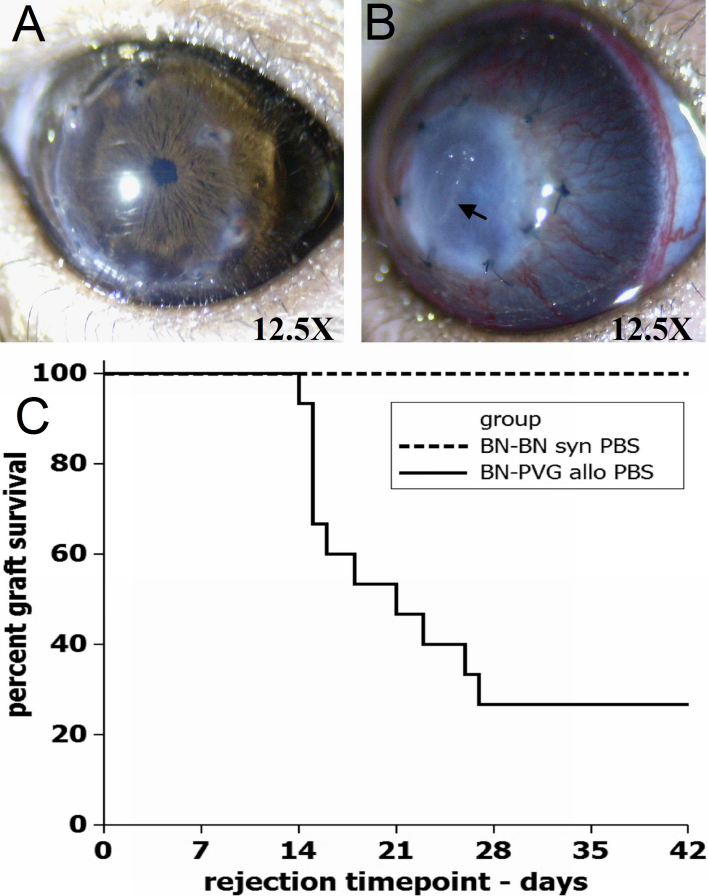
Rejection kinetics of corneal allografts in the BN-PVG strain combination. **A**: An example of a well healed corneal allograft approximately day 21. **B**: Typical appearance of a rejected PVG allograft, arrow indicates epithelial rejection line (occasionally observed). **C**: Rejection kinetics of the BN-PVG strain combination BN-PVG n=15, BN-BN n=9. Kaplan–Meier-Survival plot.

Figure 1 summarizes the basic parameters for the newly developed cornea transplant model. PVG allo-grafts are rejected between day 14 and 27 post surgery (mean=18.64+/−4.8 SD; median=16 excluding long-term survivors; median=21 including long-term survivors). Approximately 25% of all grafted corneas are spontaneously accepted. Fibrin deposits were common post-operatively but resolved spontaneously after four days. Neovascularisation originating from the limbus was very pronounced and typically reached the graft by day seven to nine post-operation day (POD). Occasionally, we observed endothelial and epithelial rejection lines (Figure 1B) during the rejection process.

### FACS analysis of draining lymph nodes

#### Quantitative analysis of lymphocyte populations

**Figure 2:**
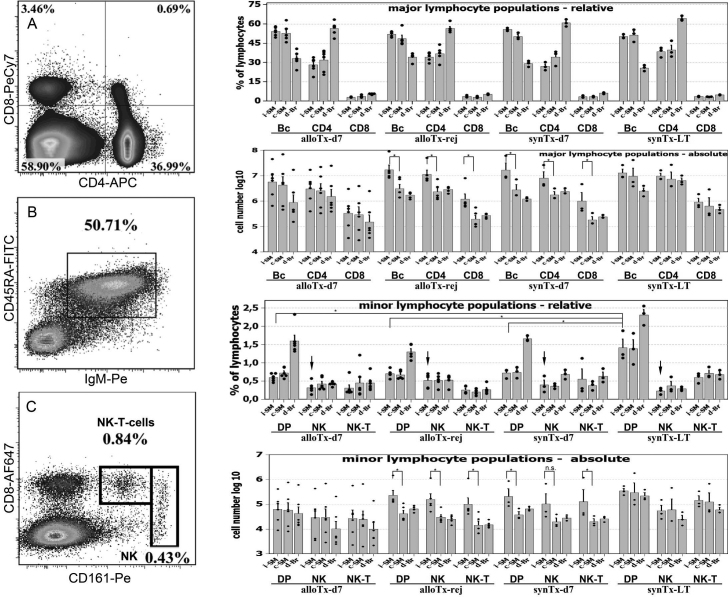
Qualitative and quantitative flow cytometric analysis of lymphocyte populations in draining lymph nodes. **A**: Representative FACS plot of CD4+ CD8+ staining used to count T-helper cells, cytotoxic T-cells and CD4+ CD8+ double positive T-lymphocytes. Events acquired: 2×10^5^. **B**: FACS plot example for B-cell detection. **C**: Representative FACS plot for NK cell assessment. NK-T cell were confirmed by CD3 expression (not shown). Bar diagrams: Cumulative results for the quantification of major and minor lymphocyte populations in draining LN of cornea transplanted animals. An asterisk (*) indicates statistical significance at p≤0.05 determined by Mann–Whitney U-Test. Allo-Tx-d7 - animals allo-grafted and analyzed at day 07 post op, n=6; allo-Tx-rej – animals displaying allo rejection of grafted corneas analyzed after the onset of rejection, n=5; syn-Tx-d7 – syngeneically grafted animals analyzed at day 7 post-op, n=3; syn-Tx-LT – syn-grafted long-term survivors analyzed at the end of the observation period at day 42; n=3.

We analyzed actively allo-rejecting animals and found that the ipsi-lateral submandibular (i-SM) lymph node (LN) was swollen and discolored (alloTx-rej, n=5). Using a multicolor staining approach we measured the cellular composition of that draining ipsi-lateral-SM LN. Moreover, we calculated the relative and absolute cell numbers of minor and major lymphocyte populations and compared these results with the contra-lateral submandibular (c-SM) LN. We also chose a distal contra-lateral brachial (d-Br) LN as reference to control for the possibility that the contra-lateral LN participates in the allo-rejection due to its close proximity to the primary draining lymph nodes. We performed this procedure on lymphatic tissue isolated from rejecting animals as well as from syngeneically grafted long-term survivors (day 42, synTx-LT, n=3). Additionally we analyzed an earlier time point (POD-07) from allo- and syn-grafted rats (alloTx-d07, n=6 or synTx-d07, n=3, respectively). First, from the data presented in Figure 2 (upper bar diagrams) one can deduce that the submandibular LNs in general have a strikingly different cellular composition than distal brachial LNs. B-cells are the dominant cell population which can be seen in all study groups. We routinely find elevated cell numbers in the draining ipsi-lateral LN (i-SM) of transplanted animals compared to the contra-lateral side due to swelling, although no discernable difference between the groups alloTx-d07, alloTx-rej, and synTx-d07. From these data we conclude that changes in lymphocyte composition are largely a result of the surgical trauma and the local inflammation that ensued afterwards, regardless of the MHC status of the graft. Concomitant to the analysis of major lymphocyte compartments the data on minor lymphocyte populations is shown in Figure 2, lower bar diagrams. In relative terms we observed a reduced frequency of CD4+CD8+ double positive T-cells (DP) in allo-recipients (alloTx-d07 and alloTx-rej) and syngeneically grafted animals on POD-07 compared to syngeneic long-term survivors (Figure 2 3rd bar diagram from top). Further a small increase in NK cell percentages is noticeable in actively rejecting study animals (arrows in Figure 2). There is, however, no difference between allo- and syngeneically grafted animals on POD-07.

#### T-cell activation markers

**Figure 3:**
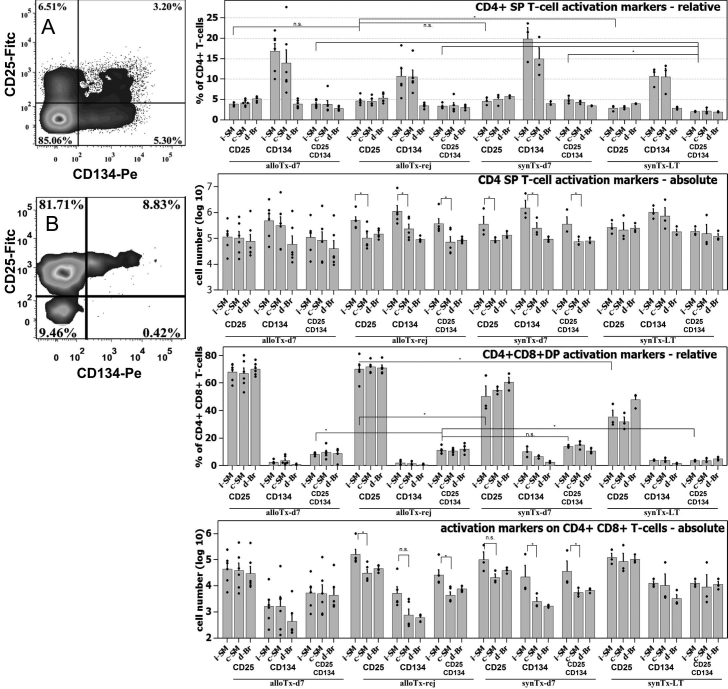
Flow cytometric analysis of helper T-cell activation markers in draining lymph nodes. **A**: Representative FACS plot of activation marker expression on CD4+ single positive T-helper cell in draining LN. Events acquired: 2×10^5^. **B**: Example of CD25 and CD134 expression pattern on CD4+CD8+ double positive T-cells. Bar diagrams: Cumulative results for the quantification of T-cell activation status of Helper T-lymphocytes. An asterisk (*) indicates statistical significance at p≤0.05 determined by Mann–Whitney U-Test.

To identify T-cell activation characteristics for allo-graft rejection we screened T-lymphocyte subpopulations (CD4+ SP and CD4+CD8+ DP) for the expression of activation markers CD25 and CD134 (Ox-40). Comparing syn-grafted long-term survivors (synTx-LT) with early post-op groups (POD-07) and rejecting animals reveals a modest relative increase in CD25 and CD25+CD134 co-expression in CD4+ single positive cells for the latter three groups (synTx-d07, alloTx-d07, and alloTx-rej, respectively; Figure 3 top bar diagram). CD134 cell surface density is elevated on cells in draining ipsi-lateral LN (i-SM) of alloTx-d07 rats compared to actively rejecting animals. However, the same relative increase in CD134 expression is observed in synTx-d07 control animals (Figure 3, upper bar diagrams). Further, we used the same cell surface marker combination to analyze the small subset of CD4+CD8+ DP T-cells and find statistically significant differences between allogeneically and syngeneically grafted animals (Figure 3, lower diagrams). A much stronger CD25 expression can be observed on allo-grafted animals at POD- 07 and the day of rejection in contrast to iso-grafted animals on POD-07 and long-term survivors. Moreover, we saw an increase of CD25 CD134 double expression in actively rejecting study subjects compared to syngeneically grafted animals at the end of the observation period. In summary some evidence for an allo-specific immune response was collected.

#### FACS analysis of graft infiltrating lymphocytes

**Figure 4:**
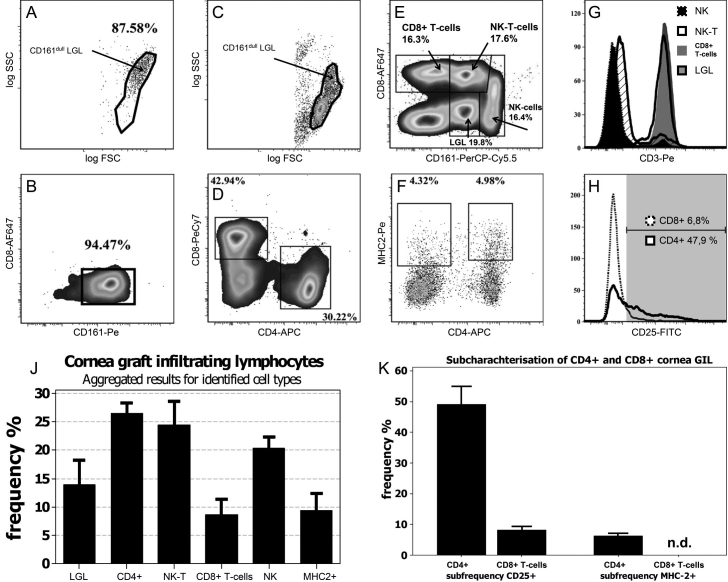
Flow cytometric analysis of graft-infiltrating lymphocyte populations. **A**, **B**: Allo-grafted cornea analyzed at day 7 post-op. **A**: Forward-sideward scatter morphology of graft infiltrating cells. **B**: Representative image of CD161^dull^ expression on LGL. No other cell type could be detected. Recorded events: 2–5×10^3^. **C**-**H**: FACS results of allo-rejecting corneas: Events acquired: 5×10^3^ to 3×10^4^ per sample. **C**: FSC-SSC morphology of GIL. **D**: Detection of T-lymphocytes. **E**: Measurement of CD8 and CD161 NK markers on GIL. **F**: Sub-characterization of CD4+ T-cells and MHC-2 detection. **G**: Expression pattern of CD3 from populations gated in **E**. **H**: Measurement of CD25 T-cell activation markers on populations gated in **D**. Cut-off or positive CD25 expression was determined by measuring isotype FMO samples on lymphocytes in draining LN from the same animal. For scaling reasons data are not shown in histogram. Bar diagram: Summary of all lymphocyte specimens identified in rejected corneas (left diagram) and appropriate sub-characterizations (right diagram) n=5.

To add to the comprehensive analysis of the BN-PVG graft rejection process, we also studied lymphocyte populations which infiltrated the allograft (Figure 4). For that purpose we developed a gentle digestion procedure to quickly isolate lymphocytes from transplanted corneas. By performing multi-parameter flow cytometry on individual corneal preparations we were able to identify six distinct cell populations infiltrating a rejecting graft in this model. We found that CD4+ T-cells represent the largest single fraction, of which approximately 50% are CD25 positive and a small subpopulation of CD4 and MHC-2 positive cells. Further, we can detect CD8+ T-cells as well as CD8+ CD161^dull^ NK-T-cells and CD8+/− CD161^high^ CD3- NK cells. The latter two cell-types constitute almost 50% of all GIL. Additionally, we observed a large granular cell type with CD161^dull^ expression. These cells did not stain MHC-2 positive (determined by back-gating strategy) and could already be detected at POD-07 in allogeneic and syngeneic grafts (Figure 4A,B).

#### Serum analysis

**Figure 5:**
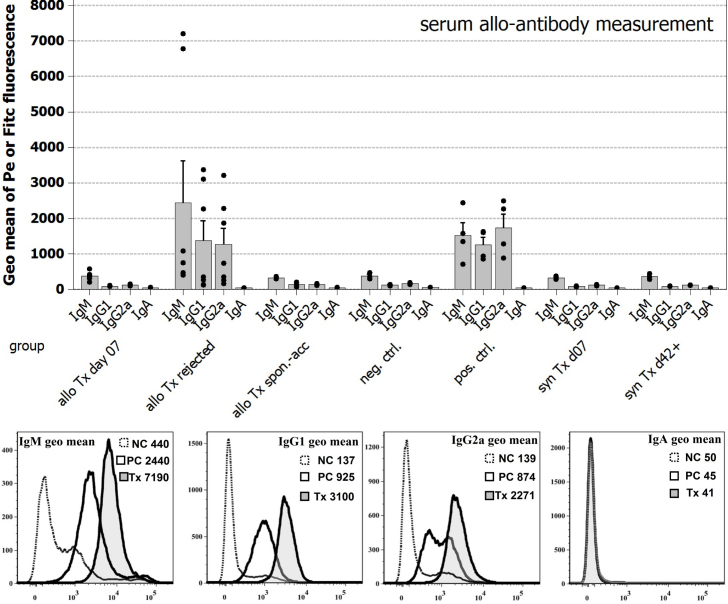
Serum allo-antibody measurement in grafted animals. Bar diagram: Summary of allo-antibody screening in transplanted animals using a FACS based direct detection method. The presence of IgG1 infers a Th2 response, while the presence of IgG2a antibodies indicates a Th1-IFN-γ induced class switch in B-cells. For IgM detection IgM+ B-cell were excluded from analysis by CD45RA staining (see Figure 2B); IgA isotypes could not be detected. FACS plots: Display of the measurement method for direct binding of allo-antibodies to allogeneic target cells (PVG donor splenocytes); negative controls: serum from PBS injected animals harvested after day 14, positive controls: serum from animals injected with 1×10^6^ γ-irradiated thymic dendritic cells collected after day 14.

To complete the comprehensive analysis of the model we collected serum from all animals in the study and screened it for the presence of allo-antibodies. As Figure 5 shows, we find evidence for the presence of anti-PVG IgM, IgG1, and IgG2a immunoglobulins which directly bind allogeneic target cells (PVG-splenocytes). Allo-transplanted animals analyzed at POD-07 or graft acceptors did not exhibit anti-donor antibody responses.

## Discussion

We find the BN-PVG rat strain combination to be an exceptionally robust model with little intra- and post-operative complications, which makes it an ideal choice for pre-clinical studies. Furthermore, we find that this model with its delayed rejection kinetics, rate of spontaneous acceptance and occurrence of rejection lines more closely mimics the clinical situation in humans (Figure 1) [14].

### Analysis of draining lymph nodes

We found that the ipsi-lateral submandibular LN was inflamed as a result of allogeneic penetrating keratoplasty. However, signs of inflammation in the draining submandibular LN are largely indistinguishable from inflammation caused by the surgical trauma in syngeneic recipients and the resulting breach of the immunological barrier of the eye (Figure 2). To detect allo-primed T-cells we chose classical activation markers CD25 and CD134 because they perform excellently when studying in-vitro cultures of allo-activated T-cells (M.M. and T.R., unpublished). We expected a certain amount of T-cell activation background due to normal immunological processes in non-sterile animals and regulatory T-cells with a persistent activated phenotype, hence our choice to focus on an inflamed lymph node rather than the spleen and blood. Contrary to our expectations we find no unambiguous pattern of allo-specific activation in draining LN (Figure 3). From that we conclude, that the number of allo-antigen-specific T-cells is dwarfed by bystander activation of helper T-cells due to the surgical trauma and the normal background of activated T-lymphocytes due environmental antigens. Some evidence for an allo-specific immune response stems from the observation of increased frequencies and absolute numbers of CD4+CD8+ double positive T-cells in ipsi-lateral draining LN of allo-grafted recipients. CD8alpha expression on CD4+ T-cells is not observed in mice. It is however a feature of rat and human CD4+ T-lymphocytes and is considered as an activation marker [15,16]. Kenny et al. [17] determined that this peculiar subpopulation is dominated by Th1 helper T-cells. Given the clear difference seen between allo- and syn-grafted animals with regard to CD25 expression in CD4+CD8+DP cells, we hypothesize that truly allo-antigen-specific T-cells may be found in that compartment.

### Allo-antibody serum analysis

Despite the obvious signs of an immune response, the graft rejection, we aimed at establishing more parameters by which an allo-antigen driven immune reaction can be detected. We found that a serum analysis accomplishes this task. Since spontaneous graft accepters do not form allo-antibodies a serum analysis is a good proxy parameter for the allo-rejection process. Particularly intriguing is the observation of anti-donor IgG2a antibodies. Despite the strong apparent Th2-type bias we inferred from the massive presence of B-cells in the draining submandibular LN and the presence of IgG1 isotypes, a Th-1 immune response must have occurred, which provided interferon-γ for the antibody class switch from IgG1 to IgG2a [18]. In contrast to other cell types B-cells and allo-antibodies do not garner much attention in the context of allo-graft rejection. Complement and antibodies can be detected in human aqueous humor [19], and allo-antiserum from rejecting animals has been found to confer complement dependent as well as independent cytolysis [20,21]. Interestingly, antigen-presenting B-lymphocytes also appear to play a significant role in the maintenance of the immune-privileged status of the cornea by inducing CD8+ suppressor T-cells [22].

### Graft infiltrating lymphocytes

Collagenase digestion as a method to isolate lymphocytes has been used previously in mouse studies of inflamed corneas [23]. However, the technique has not been applied on allo-grafted tissue so far. FACS measurements of the corneal rejection process have so far only been performed on aqueous humor samples in rat and human [10,24]. Using our rapid digestion protocol for corneas and the subsequent FACS analysis of isolated cells we can confirm the presence of CD25 positive and negative CD4+ helper T-cells and CD8+ cytotoxic T-lymphocytes. Moreover, we could demonstrate that the often observed NK-marker CD161 [10,11] is expressed on three distinct cell populations. In fact the majority of CD8+ expressing cells are either NK-T-cells or CD3- NK cells. We further identified a cell population with a CD161^dull^ phenotype, which is already present at day POD-07 in syngeneically and allogeneically grafted animals. Due to this observation it is possible that previous IHC studies have overestimated the number of infiltrating natural killer cells. We assume these cells to activated monocytes as described by Scriba et al. [25]. Whether these cells are identical to ED2+ macrophages as reported by Larkin et al. [9] remains to be determined. Monocytes and macrophages are of particular interest and importance in the uptake of allogeneic material and the initiation of an allo-antigen-specific immune response. It has been shown that, when monocyte infiltration into the graft is blocked by a toxic drug [26] allo-graft rejection can be completely prevented.

Except for the few instances where CD161+ cells were isolated from aqueous humor and were shown to have lytic capacities against allogeneic target cells [10], functional assays on GIL have not been performed so far. Our novel isolation method of GIL allows FACS sorting of viable lymphocytes and might contribute greatly in answering the question of the precise graft destruction process. In general our findings of graft infiltrating lymphocytes are in good agreement with results previously obtained by IHC [9-11,27]. A putative limitation of this experimental approach should be mentioned though. We can not exclude the possibility that strongly adherent cells such as Langerhans cells and macrophages are not isolated in sufficient numbers. Thus, our results obtained might be skewed toward higher frequencies of non-adherent lymphocytes.

To conclude, our results demonstrate that despite its immune privileged status and the low-responder characteristics of the strain combination, allogeneic corneal grafts mount a full fledged Th1 and Th2 immune response similar to high responder strain combinations. The idiosyncrasies of different strain combinations with regard to rejection speed are not understood in general. The immune response that we measured in the BN-PVG strain combination appears to be normal. With the exception of T-cell activation pattern, for which there is no comparable data set, the different facets of the allo-rejection process that we describe here appear to be identical to high responder strain combinations.

The adaptive immune response against allogeneic corneal tissue has been well established in the shape of indirectly primed allo-antigen specific CD4+ helper T-cells [7,28]. However, CD4 knockout mice still reject allogeneic corneas, albeit in a delayed fashion [29]. Furthermore, baby rats with an immature adaptive immune system readily reject fully mismatched corneal grafts and the accelerated rejection is accompanied by increased numbers of graft infiltrating NK and NK-T-cells [30]. The existence of alloreactive NK and NK-T-cells has been established in rat and human cornea transplant settings [10,24]. We hypothesize that the presence of NK-T-cells and NK-cells in rejecting corneas shows the synergy between innate and adaptive immunity during allograft destruction, whereby the adaptive arm of the immune system triggers and boost the host immune reaction but the actual graft destruction is mediated by alloreactive NK and NK-T-cells according to the ‘missing self hypothesis’ [31].
